# Ping-Chong-Jiang-Ni Formula Induces Apoptosis and Inhibits Proliferation of Human Ectopic Endometrial Stromal Cells in Endometriosis via the Activation of JNK Signaling Pathway

**DOI:** 10.1155/2017/6489427

**Published:** 2017-06-01

**Authors:** Rui-Ning Liang, Pei-Shuang Li, Yang Zou, Yu-Ling Liu, Zhen Jiang, Zhen Liu, Pei Fan, Ling Xu, Jia-Hua Peng, Xue-Yan Sun

**Affiliations:** ^1^Jiangxi University of Traditional Chinese Medicine, Nanchang, Jiangxi 330004, China; ^2^Department of Gynecology, The Affiliated Hospital of Jiangxi University of Traditional Chinese Medicine, Nanchang, Jiangxi 330006, China; ^3^Key Laboratory of Women's Reproductive Health of Jiangxi Province, Jiangxi Provincial Maternal and Child Health Hospital, Nanchang, Jiangxi 330006, China; ^4^Department of Gynecology & Obstetrics, Yingtan Hospital of Traditional Chinese Medicine, Yingtan, Jiangxi 335000, China; ^5^Department of Gynecology & Obstetrics, Nankang Maternal and Child Health Hospital, Nankang, Jiangxi 341400, China; ^6^Department of Gynecology & Obstetrics, The Second Affiliated Hospital of Jiangxi University of Traditional Chinese Medicine, Nanchang, Jiangxi 330012, China

## Abstract

Endometriosis is a common gynecological condition in childbearing age women and its therapy in modern medicine achieves usually temporary cure. Ping-Chong-Jiang-Ni formula (PCJNF), a Chinese herbal medicine (CHM), was shown to be clinically effective on endometriosis. Meanwhile, c-Jun N-terminal kinase (JNK) signaling pathway was involved in the therapeutic process of CHM on endometriosis. Here, we explored the effect of PCJNF on the ectopic endometrial stromal cells (EESCs) from endometriosis and test whether JNK signaling was involved. After being treated with PCJNF-containing serum obtained from Sprague Dawley rat, cell proliferation, migration, invasion, and apoptosis were evaluated in EESCs, and the total and phosphorylated JNK, ERK, and p38 proteins were detected. Our results showed that PCJNF could suppress cell proliferation, migration, and invasion and induce apoptosis in EESCs. The suppressed proliferation and increased apoptosis were dependent on JNK activation. Additionally, PCJNF caused cell cycle arrest at G2/M phase and this effect was mediated by JNK signaling, while the decreased cell migration and invasion treated by PCJNF were independent of JNK signaling. In summary, our results provided the first evidence that PCJNF could suppress cell proliferation, migration, and invasion, while increasing apoptosis in EESCs, and the suppressed proliferation and enhanced apoptosis were mediated by JNK signaling.

## 1. Introduction

Endometriosis is a common benign disorder characterized by the ectopic growth of endometrium, affecting approximately 15% of women of reproductive age, causing chronic pelvic pain, dysmenorrheal, irregular menstrual cycle, and infertility [1]. The common therapy strategies for this disorder are involved in hormone therapy and surgical removal of endometriotic lesions, depending mainly on the patients' symptoms and desire of future fertility [2, 3]. However, these therapeutic strategies could only achieve a temporary cure; it is thus urgent to search for novel alternative therapy for this disease.

Endometriosis exhibits several features such as aberrant growth and metastasis; the development of this disorder includes a course of cell proliferation, apoptosis, invasion, and migration [4, 5]. Among which, the disrupted balance between cell proliferation and apoptosis are crucial for the development of endometriosis; furthermore, the enhanced cell invasion and migration are also crucial for the development of this disorder [6, 7]. On the other hand, the endometrial tissue contains mainly epithelial and stromal components, among which, stroma supports and provides a regulatory role for the development and differentiation of the overlying epithelium. Furthermore, increasing studies have also suggested that endometrial stromal cells (ESCs) play essential roles in the initiation and development of endometriosis [7, 8]. For example, the stromal cells, rather than the epithelia cells, can be affected by the estradiol and progesterone, suggesting a crucial role of stromal cells played in the endometrial functions [7, 8]. It thus attracted increasing attention to explore the possible therapeutic effect of alternative therapy via targeting ESCs of endometriosis.

Accumulated evidences have shown that CHM possessed good therapeutic effects on multiple human diseases [9–11]. Also, multiple Chinese formulas have been used to treat endometriosis [1, 12–15]. Among which, PCJNF, a Chinese herbal formula, composed of eight herbs including* Ramulus Cinnamomi*,* Sanguis Draconis*,* Faeces Trogopterori*,* Pollen Typhae*,* Chinese Eaglewood*,* Whitmania pigra *Whitman,* Liriope spicata*, and* Glycyrrhiza uralensis *Fisch, had been shown to have good therapeutic effect on endometriosis in our multicentered clinical trial research [16], where we enrolled a total of 113 women with moderate/severe endometriosis and gave them PCJNF each day, continuous for 3 months without intervals; our results showed that the ratio of dysmenorrheal relief in these women was 93.81% (106/113), and the serum levels of cancer antigen 125 (CA125) and prolactin (PRL) decreased significantly after the administration.

The JNK signaling pathway can be activated by multiple stress stimuli, including drug treatments and cellular stresses, and involved in the adaptation to these stresses [17–20]. The molecular mechanisms of Traditional Chinese Medicine (TCM) therapy in human diseases usually included activation and deactivation of diverse signaling pathways [21–23], and JNK signaling pathway was found to be frequently involved in the process of TCM therapy in diverse diseases, including endometriosis [2, 24]. In the present study, we attempt to explore whether there is actual effect of PCJNF on EESCs in endometriosis and whether JNK signaling pathway is involved in the therapeutic process.

## 2. Materials and Methods

### 2.1. Animals and Ethics Statement

Forty healthy female SD rats weighing 200 ± 25 g were obtained from the Experimental Animal Center of Jiangxi University of Traditional Chinese Medicine. They were kept under specific pathogen-free conditions and fed with a standard diet and given water ad libitum, in accordance with the guidelines of the association for Assessment and Accreditation of Laboratory Animal Care, and all procedures were approved by the Animal Care and Use Committee of Jiangxi University of Traditional Chinese Medicine.

### 2.2. Preparation of Physiological Saline Control and PCJNF-Containing Serums

PCJNF was obtained from the Affiliated Hospital of Jiangxi University of Traditional Chinese Medicine (Nanchang, Jiangxi, China). An extract of PCJNF was obtained by decocting the dried prescription of herbs (15 g* Ramulus Cinnamomi*, 3 g* Sanguis Draconis*, 10 g* Faeces Trogopterori*, 6 g* Pollen Typhae*, 6 g* Chinese Eaglewood*, 3 g* Whitmania pigra* Whitman, 10 g* Liriope spicata*, and 6 g* Glycyrrhiza uralensis* Fisch) in 12 volumes of water (v/w) for 30 minutes and extracted twice; the suspension was collected by filtration and condensed to a concentration of 1.82 g/ml solution and stored at 4°C for experimental use. The dose of 18.2 g/kg once per day was equal to that of clinical used.

After 5 days of acclimation, these female SD rats were randomly divided into a PCJNF group (*N* = 20) and a physiological saline control group (*N* = 20). Rats were treated for 2 weeks: each rat in the PCJNF group was orally given PCJNF extract at doses of 18.2 g/kg twice per day for 7 successive days, respectively. The physiological saline control group received the equal volume of saline within the same time. After 2 hours of the final administration, rats were anesthetized with pentobarbital sodium and the blood samples were collected through the abdominal aorta. The serums were collected by centrifugation at 3,000 rpm for 10 minutes at 4°C. The serums were then isolated and subject to a 0.22 *μ*m Millipore filter, stored at −80°C until use.

### 2.3. Cell Isolation and Culture

Human ectopic endometrial tissues during the luteal phase of the menstrual cycle were obtained from endometriosis samples who received no hormone treatment for >3 months, according to the guidelines of the Declaration of Helsinki, after informed consent had been obtained and with approval by the Institutional Review Board from Jiangxi University of Traditional Chinese Medicine. A total of 2 endometrial samples (35- and 42-year-old women) undergoing surgical resection were recruited and the ectopic endometrial tissues were minced into small pieces and incubated with 1 mg/mL type IV collagenase dissolved in complete culture medium for 2 hours at 37°C; the resultant digest was filtrated and centrifuged at 2,000 rpm for 10 minutes; the obtained pellets were collected and grown in complete culture medium, as described previously [5, 25]. The isolated primary EESCs were cultured in DMEM/F12 with 10% FBS, containing 100 IU/mL penicillin and 50 mg/mL streptomycin. Cells were treated with DMSO, saline control serum (NS), NS plus the selective JNK inhibitor SP600125 (300 *μ*M, dissolved in DMSO), PCJNF serum (PCJNF), and PCJNF plus JNK inhibitor SP600125 (300 *μ*M, dissolved in DMSO) 48 h prior to the experiment, respectively.

### 2.4. Immunocytochemistry

Cells seeded on 6-well chamber slides were firstly fixed with 4% paraformaldehyde; 0.75% H_2_O_2_ dissolved in methanol was used to block the endogenous peroxidase activity for 15 minutes at room temperature. Cell characterization of the isolated cells were evaluated by using immunostaining of mouse anti-vimentin (sc-73258, 1 : 500) (Santa Cruz Biotechnology, Santa Cruz, CA, USA) and cytokeratin (sc-53264, 1 : 500) (Santa Cruz Biotechnology, Santa Cruz, CA, USA). The purity of the isolated EESC was assessed with light microscopy.

### 2.5. Immunoblot Analysis

Cells were lysed by NP-40 with protease inhibitors and phosphatase inhibitors; the proteins were separated via SDS-PAGE electrophoresis, transferred onto nitrocellulose membranes, and then incubated with primary and secondary antibodies, respectively. Primary antibodies against JNK1/2/3 (YT2441) (1 : 1000) and phospho-JNK1/2/3 (phospho Thr183/Y185) (1 : 1000) were obtained from ImmunoWay Biotechnology (Newark, DE, USA). Primary antibodies against ERK (ab196883, 1 : 1000), p-ERK (ab50011, 1 : 5000), p38 (ab27986, 1 : 1000), and p-p38 (ab45381, 1 : 5000) were purchased from Abcam (Abcam, Cambridge, MA, United States). The JNK inhibitor SP600125 was obtained from Santa Cruz Biotechnology (Santa Cruz, CA, USA). The immunoreactive bands were detected with an enhanced chemiluminescence detection system from Pierce (Thermo Fisher Scientific Inc., Rockford, IL, USA). Protein band was analyzed with Quantity One software (Bio-Rad Laboratories, Life Science Research, CA, USA). Protein levels were normalized to that of the internal control *β*-actin purchased from Abcam (Cambrige, MA, USA) (ab8226, 1 : 1000).

### 2.6. Cell Proliferation Assays

The Cell Counting Kit-8 (CCK8) assay was used to assess cell proliferation according to the manufacturer's instructions (Dojindo Laboratories, Tokyo, Japan). Briefly, 2 × 10^3^ cells/well were seeded in 96-well plates, and 10 *µ*L of CCK8 reagent was added to each well. After incubation of 3 hours, optical density (OD) value was determined with a spectrophotometry at wave length of 450 nm. Each assay group was performed in triplicate.

### 2.7. Transwell Cell Migration and Invasion Assays

The transwell assays were used to evaluate the migrative and invasive capacities of EESCs. The transwell chambers with 8 *μ*m pores were obtained from Corning (Corning, NY, USA). Aliquots of 2 × 10^3^ cells in serum-free DMEM (100 *μ*L) were seeded into the upper compartments of the 24-well plate; the lower chambers were filled with DMEM containing 10% FBS. After 24 hours of incubation, the migrated cells were fixed with methanol, stained with crystal violet, and counted under a light microscope (Olympus IX71, Japan). An average of five visual fields was examined. For the invasion assay, the upper chamber was precoated with 60 *μ*L Matrigel (1 : 8 dilution; BD Bioscience San Jose, CA, USA) and performed the same protocol as described above.

### 2.8. Cell Apoptosis Assay

Cell apoptosis was evaluated using Annexin V-fluorescein isothiocyanate (FITC) Apoptosis Detection Kit (556547, BD Biosciences, Franklin Lakes, NJ, USA) with flow cytometry (FCM). Briefly, cells were pretreated with JNK inhibitor (SP600125, 300 *μ*M, dissolved in DMSO) for 1 hour and then treated with PCJNF-containing serums. After 24 hours, cells were stained with 5 *μ*L FITC-conjugated Annexin V and 5 *μ*L propidium iodide (PI) for 15 minutes in the dark. Cell apoptosis was analyzed by Cytomics™ FC500 flow cytometer (Beckman Coulter, Miami, FL, USA). A collection of 10,000 events were analyzed in three independent experiments.

### 2.9. Cell Cycle Analysis

EESCs were treated with PCJNF and different controls for 48 h in complete medium. The cells were trypsinized into single cells and then fixed in 70% (v/v) ethanol at 4°C overnight. After centrifugation, cells were washed with cold PBS and stained with propidium iodide (PI) according to the manufacturer's protocol with minor revision. After incubation for 30 min in a dark room, cell cycle distribution was analyzed by flow cytometry (Beckman Coulter, Miami, FL, USA) using MultiCycle software (Beckman Coulter).

### 2.10. Statistical Analysis

All data were presented as mean ± SD. Statistical analysis was performed using SPSS version 18.0 (SPSS Inc., Chicago, IL, USA). Student's *t*-test was used for comparisons between the two groups, and one-way ANOVA was used for multiple comparisons. *P* values < 0.05 were considered statistically significant.

## 3. Results

### 3.1. Primary EESC Isolation and Characterization

**Figure 1:**
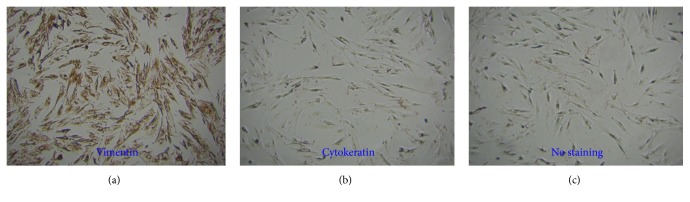
Morphological and molecular characterization of EESCs with inverted microscope and immunocytochemistry. The morphology of primary EESCs isolated from ectopic endometria of samples with endometriosis (×100) (a). Vimentin (b) and cytokeratin (c) were positively and negatively expressed in primary EESCs (×100), respectively.

The primary EESCs were stained positive with anti-vimentin, a specific marker of stromal cells [5, 26], while they were stained negative with anti-cytokeratin by immunocytochemistry, a specific molecular marker for epithelial cells (Figure 1). The purity of isolated EESCs was >95% and cells between passages 4 to 12 were adopted in the current study.

### 3.2. PCJNF Suppressed Proliferation of EESCs through Activation of p-JNK

**Figure 2:**
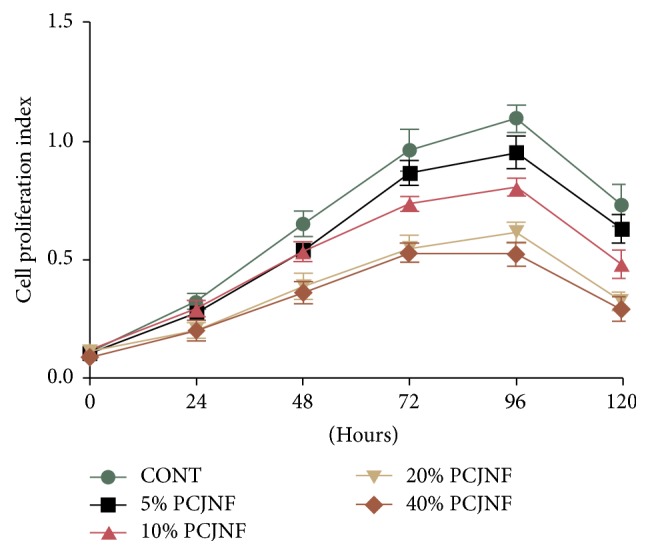
PCJNF suppressed cell proliferation in a concentration-dependent manner in EESCs. EESCs were exposed to different concentration of PCJNF-containing serum (5%, 10%, 20%, and 40%) for 0, 24, 48, 72, 96, and 120 h, respectively. The cell viability was assessed by CCK8 assay. Compared with the CONT group, PCJNF-containing serum suppressed cell proliferation in a concentration-dependent manner: 5% and 10% PCJNF-containing serum had low or medium suppressive effects on cell proliferation, while 20% and 40% PCJNF-containing serum resulted in significantly suppressed proliferation in EESCs. The experiment was performed in triplicate. CONT: EESCs cultured with complete medium. Data are presented as means ± SD of three independent tests.

**Figure 3:**
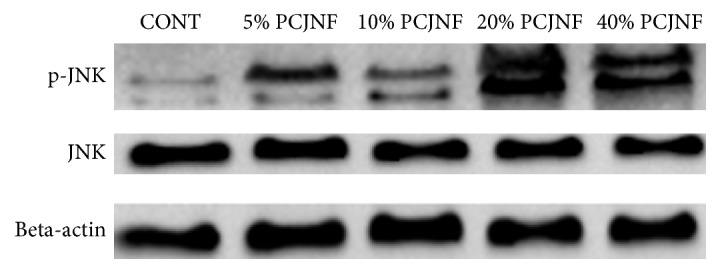
PCJNF induced the activation of JNK signaling pathway in EESCs. EESCs were exposed to different concentration of PCJNF-containing serum (5%, 10%, 20%, and 40%). Representative Western blot of beta-actin, total JNK, and phospho-JNK was shown. Different concentration of PCJNF-containing serum increased the expression of p-JNK but not the total JNK, indicating the activation of JNK signaling pathway. Compared with CONT group, 5% and 10% PCJNF-containing serum had certain effect on the activation of JNK pathway, while 20% and 40% PCJNF-containing serum resulted in obvious activation of JNK pathway. The experiment was performed in triplicate. CONT: complete medium.

**Figure 4:**
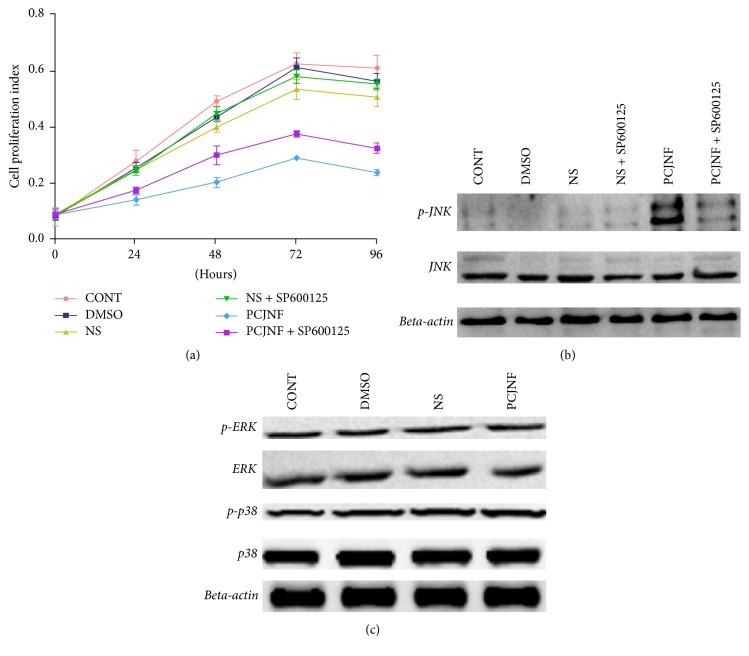
The suppressive effect of PCJNF-containing serum was mediated by JNK signaling pathway in EESCs. (a) EESCs were treated with 20% PCJNF-containing serum and different controls for 0, 24, 48, 72, and 96 h, respectively. The cell viability was assessed by CCK8 assay. Compared with different control groups (CONT, DMSO, and NS), 20% PCJNF-containing serum had significant suppressed effect on cell viability after 24 h, while the JNK inhibitor SP600125 attenuated this effect only after 48 h. The experiment was performed in triplicate. (b) Meanwhile, JNK inhibitor SP600125 deactivates the JNK signaling pathway in EESCs, when compared with different controls (CONT, DMSO, and NS). While there was no significant difference of cell proliferation in EESCs between the “NS” and “NS + SP600125” group (*P* > 0.05). (c) The total and phosphorylated ERK and p38 of EESCs did not change markedly, when compared with different controls. The experiment was performed in triplicate. NS: rat serum from saline treatment; PCJNF: rat serum with PCJNF treatment.

EESCs were exposed to culture medium containing 5%, 10%, 20%, and 40% (v/v) concentrations of PCJNF-containing serum, respectively, to determine the potential suppressed effect and activation of JNK signaling pathway. Our results showed that 20% and 40% of PCJNF-containing serum had the best suppressed effect on cell proliferation, when compared with the untreated cells (CONT), while cell proliferation decreased after 120 hours of PCJNF-containing serum treatment in EESCs (Figure 2). Meanwhile, the level of p-JNK treated with 20% and 40% PCJNF-containing serum resulted in an obvious activation of JNK pathway when compared with several lower concentration (5% and 10%) and NS (Figure 3). We thus adopt 20% concentrations of PCJNF-containing serum in subsequent cellular functional assays. Noted that, for 6-hour pretreatment with the JNK inhibitor SP600125, it could significantly attenuate cell proliferation suppression induced by PCJNF-containing serum (Figure 4(a)) and resulted in the decrease of p-JNK level (Figure 4(b)), suggesting that PCJNF-containing serum suppressed cell proliferation of EESCs through activation of p-JNK. Furthermore, the total and phosphorylated ERK and p38 in PCJNF-treated EESCs were not affected markedly when compared with that of different controls (Figure 4(c)).

### 3.3. PCJNF Induced Cell Apoptosis of EESCs through Activation of p-JNK

**Figure 5:**
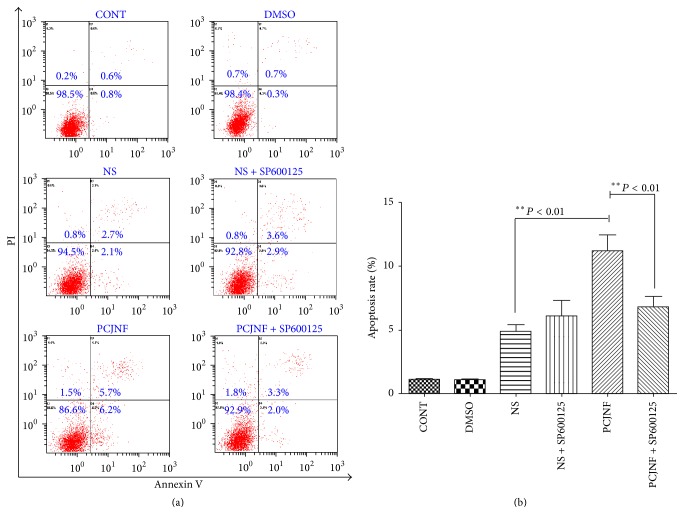
PCJNF induced cell apoptosis and this effect was dependent on the activation of JNK signaling pathway in EESCs. (a) Compared with different control group (CONT, DMSO, and NS), the percentage of apoptotic cells treated with PCJNF-containing serum increased significantly (*P* < 0.01), and JNK inhibitor SP600125 could significantly abrogate this effect (*P* < 0.01). There was no significant difference of cell apoptosis between the “NS” and “NS + SP600125” group (*P* > 0.05). (b) Analysis of apoptosis rates using bar graphs. Cell apoptosis was assessed by flow cytometry. Data are presented as means ± SD of three independent tests.

Cell apoptosis of EESCs induced by PCJNF-containing serum was assessed by Annexin V-fluorescein isothiocyanate (FITC) Apoptosis Detection Kit with FCM. Our results showed that 20% PCJNF-containing serum significantly increased cell apoptosis when compared with “NS” group (*P* < 0.01) (Figures 5(a) and 5(b)). In addition, the blockage of JNK signaling with SP600125 inhibitor could significantly reverse cell apoptosis treated by PCJNF-containing serum, suggesting that PCJNF induces cell apoptosis through activation of p-JNK in EESCs (*P* < 0.01) (Figures 5(a) and 5(b)).

### 3.4. PCJNF Inhibited Migration and Invasion of EESCs Independent of JNK Signaling Pathway

**Figure 6:**
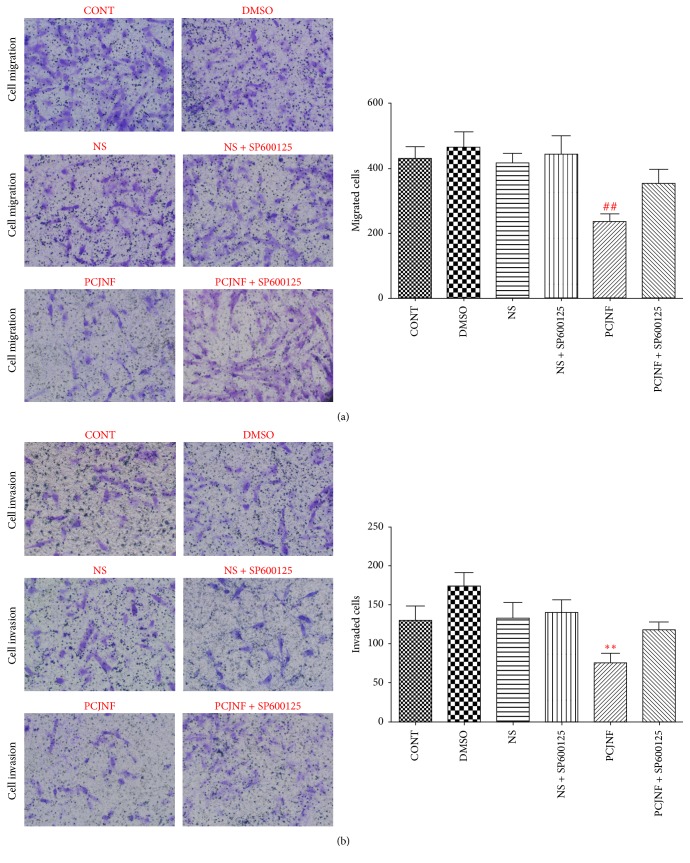
PCJNF decreased cell migration and invasion, while this effect was independent of the activation of JNK signaling pathway in EESCs. EESCs were exposed to different control groups (CONT, DMSO, and NS) and 20% PCJNF-containing serum, respectively. Compared with different control group (CONT, DMSO, and NS), 20% PCJNF-containing serum inhibited significantly the cell migration (*P* < 0.01) (a) and invasion (*P* < 0.01) (b) capacities in EESCs, respectively. Furthermore, JNK inhibitor SP600125 attenuated these effects obviously. Data are presented as means ± SD of three independent tests. ## and *∗∗* mean that the *p* values are less than 0.01 and the statistical differences are significant.

We evaluated the migrative and invasive effects of EESCs treated by 20% of PCJNF-containing serums with transwell assays. PCJNF-containing serums could significantly inhibit the migration (Figure 6(a)) (*P* < 0.01) and invasion (Figure 6(b)) (*P* < 0.01) of EESCs relative to that of “NS” group. However, the JNK inhibitor SP600125 did not attenuate the apoptotic level of EESCs treated with PCJNF-containing serums (*P* < 0.05). These results showed that PCJNF could inhibit migration and invasion of EESCs, while these effects were independent of JNK signaling pathway.

### 3.5. EESCs Underwent Increased G2/M Phase Arrest after PCJNF Treatment

**Figure 7:**
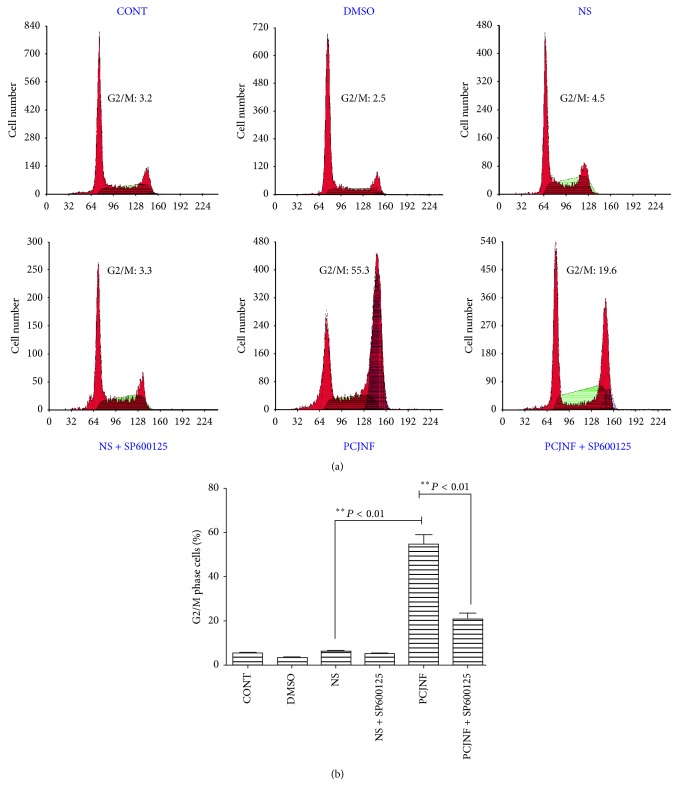
Effect of PCJNF on cell cycle distribution in EESCs. Cell cycle distribution was determined by flow cytometry. (a) PI staining assay was performed after treatment with PCJNF, PCJNF plus SP600125, and different controls, after treatment for 48 h. (b) Statistical analysis of cell cycle phase distribution. Data are presented as means ± SD of three independent tests.

To evaluate whether the inhibitory cell proliferation effect of PCJNF will affect cell cycle distribution in EESCs, we applied FCM to analyze the cell cycle distribution. At 48 h after PCJNF treatment, the percentage of G2/M phase cells was significantly increased when compared with the different controls; furthermore, the JNK inhibitor SP600125 could attenuated the G2/M arrest (Figure 7). The results suggest that treatment of PCJNF on EESCs could increase G2/M phase arrest and this effect was mediated by JNK pathway.

## 4. Discussion

TCMs and their ingredients have been regarded as the most important therapeutic agents in China for more than 2,000 years. Based on the systematic intervention strategy, TCMs are often used in a combined form which usually composed of multiple chemical components from several herbs, which usually could strengthen the therapeutic effects or minimize the potential adverse effects of certain components [27–29]. For example, we have shown that PCJNF had good therapeutic effects in endometriosis in our prior clinical study [16]. In spite of the clinical observations, the underlying molecular mechanisms remain unexplored. The present study characterized the antiproliferation and proapoptotic properties of PCJNF in EESCs in endometriosis; moreover, we also wondered whether JNK signaling pathway was involved in the therapeutic effect of PCJNF on endometriosis.

Previous study has shown that* Ramulus Cinnamomi* might induce cell apoptosis in diabetic peripheral neuropathy [30]. Similarly, we observed that PCJNF-containing* Ramulus Cinnamomi*, the king herb of this formula, also increased cell apoptosis in EESCs markedly when compared with that treated with control saline serum. Furthermore, this effect was dependent on the activation of JNK signaling pathway, which was usually involved in cell apoptosis induction under diverse stimulus. It should be noted that* Ramulus Cinnamomi* did not affect cell apoptosis in human non-small-cell lung carcinoma cell line A549 [31]; we speculated that this differential effect of* Ramulus Cinnamomi* on cell apoptosis might be cell line-specific; alternatively, the differential components between these formulas could be responsible for this discrepancy.

Previous study has shown that* Ramulus Cinnamomi* could inhibit cell proliferation in A549 lung carcinoma cell line, via inducing cell cycle arrest in G1 and G2 phases [32]. Similarly, we here showed that PCJNF inhibited significantly cell proliferation of EESCs relative to that treated with saline control serum. Here, we further showed that the suppression of cell proliferation treated with PCJNF in EESCs was JNK signaling-dependent, which showed that blockage of JNK signal could attenuate the inhibitor of cell proliferation. Furthermore, our FCM analysis results showed that PCJNF could cause significant cell cycle arrest in G2/M phase in EESCs when compared with different control groups, and JNK inhibitor SP600125 could attenuate this arrest.

Clinically, endometrial tissues might metastasize both locally and distantly and thus inhibit endometriotic cell invasion and migration might be crucial for the management of endometriosis [32–34]. Here we showed that PCJNF could effectively inhibit the migration and invasion of EESCs in vitro, suggesting that this Chinese herbal formula could be a potential alternative option for the treatment of patients with endometriosis. Furthermore, the effect of inhibitory invasion and migration effects of PCJNF on EESCs did not activate the JNK signaling pathway, implicating that some other signaling, rather than JNK signaling, was involved in the inhibitory process. It should be noted that prior studies have shown that JNK signaling pathway was involved in the process of cell invasion and migration in human disorders: it was either activated or inhibited in different cancers and/or under different extra- and intracellular stimuli [35–38]. Even though in the process of TCM therapy of human diseases, the activation or inhibition of JNK signaling pathway was inconsistent: it was activated in the decreased migration and invasion in HepG2 cells treated by the Chinese herbal formula QHF [39], while being inhibited in the inhibitory cell migration and invasion of MDA-MB-231 cells treated with the Chinese herbal formula PC-SPESII [40]; it thus seemed that the potential roles of JNK signaling pathway in cell migration and invasion were more complex than previously thought.

## 5. Conclusions

We showed that PCJNF could promote apoptosis and suppress proliferation of EESCs, at least partly, via activation of JNK signaling pathway for the first time. In addition, PCJNF could also effectively suppress cell invasion and migration in vitro, while this effect was independent of JNK signaling pathway. These results provide experimental evidences supporting the rationality of use of PCJNF in the administration of endometriosis.
